# *In vitro* effects of resistin on epithelial to mesenchymal transition (EMT) in MCF-7 and MDA-MB-231 breast cancer cells – qRT-PCR and Westen blot analyses data

**DOI:** 10.1016/j.dib.2019.104118

**Published:** 2019-06-15

**Authors:** Dimiter Avtanski, Anabel Garcia, Beatriz Caraballo, Priyanthan Thangeswaran, Sela Marin, Julianna Bianco, Aaron Lavi, Leonid Poretsky

**Affiliations:** a Gerald J. Friedman Diabetes Institute at Lenox Hill Hospital, Northwell Health, New York, NY, USA; b The Feinstein Institute for Medical Research, Northwell Health, Manhasset, NY, USA; c Donald and Barbara Zucker School of Medicine at Hofstra/Northwell, Hempstead, NY, USA

**Keywords:** Resistin, Epithelial to mesenchymal transition (EMT), Breast cancer

## Abstract

Resistin is an adipokine produced by the white adipocytes and adipose-derived macrophages, which mediates inflammation and insulin resistance Huang et al., 1997 and Renehan et al., 2008 Feb. Here, we provide data on the effect of resistin on epithelial to mesenchymal transition (EMT) in breast cancer cells *in vitro*. As model systems, we used human MCF-7 (low-metastatic) and MDA-MB-231 (high-metastatic) breast cancer cell lines. To optimize experimental conditions, we treated the cells with various concentrations of resistin (12.5, 25 and 50 ng/ml) for different time intervals (6 and 24 hours), and measured SOCS3 mRNA expression by using qRT-PCR analysis. Further, we used qRT-PCR and Western blot analyses to measure the expression of various epithelial (E-cadherin, claudin-1) and mesenchymal (SNAIL, SLUG, ZEB1, TWIST1, fibronectin, and vimentin) markers after resistin treatment. This data article is part of a study Avtanski et al., 2019 May, where detailed interpretation and discussion can be found.

**Table:** Specifications table

Subject area	*Biology*
More specific subject area	*Oncology*
Type of data	*Figures, tables*
How data was acquired	*qRT-PCR: QuantStudio 3, Applied Biosystems* *Western blot: MyECL Imager, Thermo Fisher* *Statistical analysis: GraphPad Prism software, GraphPad Software, Inc.*
Data format	*Raw and analyzed*
Experimental factors	*MCF-7 and MDA-MB-231 breast cancer cells were treated with various concentrations of resistin (8.5, 12.5, 25, and* 50 ng/ml*) in different time-points (6 and 24 hours).*
Experimental features	*Concentration-response and time-dependent optimization experiments to investigate the effect of resistin on epithelial to mesenchymal transition (EMT) in breast cancer cells in vitro.*
Data source location	New York, USA, Friedman Diabetes Institute at Lenox Hill Hospital, Northwell Health
Data accessibility	Raw data is provided
Related research article	Avtanski D, Garcia A, Caraballo B, Thangeswaran P, Marin S, Bianco J, Lavi A, Poretsky L. Resistin induces breast cancer cells epithelial to mesenchymal transition (EMT) and stemness through both adenylyl cyclase-associated protein 1 (CAP1)-dependent and CAP1-independent mechanisms. Cytokine. 2019 May; 120:155–64 [3].

**Table:** 

**Value of the data** • We demonstrated that resistin induces mRNA and protein expression of mesenchymal markers in MCF-7 and MDA-MB-231 breast cancer cells, and inhibits epithelial markers expression in MCF-7 cells. • We established optimal conditions for resistin treatment in human breast cancer cells in vitro as initial necessary step for performing further experiments. • Suppressor of cytokine signaling 3 (SOCS3) can be used as a control gene to evaluate the responsiveness of cancer cells to resistin treatment. • These data can be used to further strengthen our understanding of the role of obesity in cancer.

## 1 Data

### 1.1 Effect of resistin on suppressor of cytokine signaling 3 (SOCS3) mRNA expression

**Fig. 1:**
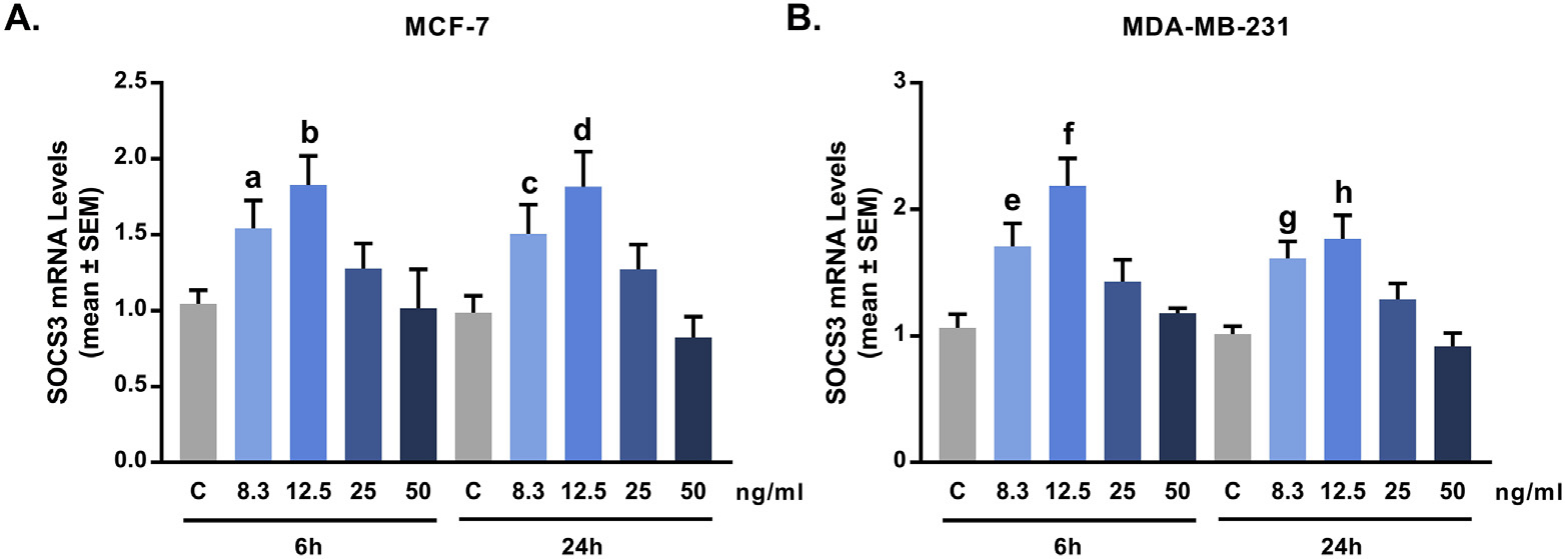
**Optimization of resistin treatment of MCF-7 and MDA-MB-231 cells.** Concentration-response and time-dependent experiments. MCF-7 (A) and MDA-MB-231 (B) cells were treated with various concentrations of resistin (8.3, 12.5, 25, and 50 ng/ml) for 6 and 24 hours. SOCS3 mRNA levels were measured by using qRT-PCR analysis. GAPDH was used as a housekeeping gene. Relative mRNA levels and p values are listed in Table 1.

Resistin is acytokine produced by the white adipose tissue (WAT) that is known to play a major role in mediating insulin sensitivity of the insulin target tissues [1], [2]. Previous research by Steppan et al. [4] demonstrated that resistin treatment of 3T3-L1 adipocytes (at concentration of 20 ng/ml) markedly induced the gene expression of suppressor of cytokine signaling 3 (SOCS3). Here, we used SOCS3 as a control gene to optimize the treatment conditions of human MCF-7 and MDA-MB-231 breast cancer cells with resistin. Cells were treated with increasing concentrations of human resistin (8.3, 12.5, 25, and 50 ng/ml) for 6 and 24 hours, and resistin mRNA expression levels were measured by qRT-PCR analysis. Data were presented as fold-change of resistin treatment *vs.* non-treated (Control) cells at 6 and 24 hours. Results demonstrated that resistin statistically significant upregulated SOCS3 mRNA levels in both, MCF-7 and MDA-MB-231 cells, as maximal effect was reached at concentration of 12.5 ng/ml for 6 hours of treatment (Fig. 1 and Table 1).

**Table 1:** **Effect of resistin on SOCS3 mRNA levels in MCF-7 and MDA-MB-231 cells.** Concentration-response and time-dependent experiment using MCF-7 and MDA-MB-231 cells treated with resistin (8.3, 12.5, 25, and 50 ng/ml) for 6 and 24 hours. SOCS3 mRNA levels were measured by qRT-PCR analysis (see also Fig. 1). *p* values < 0.05 are shown in bold.

Treatment Time	Cell Line	MCF-7		MDA-MB-231	
	Concentration (ng/ml)	mean ± SEM	p Value	mean ± SEM	p Value
**6h**	**Control**	1.047 ± 0.088		1.066 ± 0.107	
	**8.3**	1.542 ± 0.184	**0.0248**	1.706 ± 0.183	**0.0053**
	**12.5**	1.829 ± 0.192	**0.0002**	2.187 ± 0.216	**0.0001**
	**25**	1.277 ± 0.166	0.1864	1.430 ± 0.172	0.0752
	**50**	1.016 ± 0.256	0.8854	1.182 ± 0.039	0.4322
**24h**	**Control**	0.988 ± 0.112		1.017 ± 0.061	
	**8.3**	1.505 ± 0.194	**0.0353**	1.611 ± 0.136	**0.0002**
	**12.5**	1.815 ± 0.231	**0.0252**	1.769 ± 0.185	**0.0020**
	**25**	1.272 ± 0.162	0.2091	1.290 ± 0.124	**0.0549**
	**50**	0.823 ± 0.139	0.4503	0.918 ± 0.104	0.4699

### 1.2 Effect of resistin on the expression of epithelial to mesenchymal transition (EMT) markers in breast cancer cells *in vitro*

**Fig. 2:**
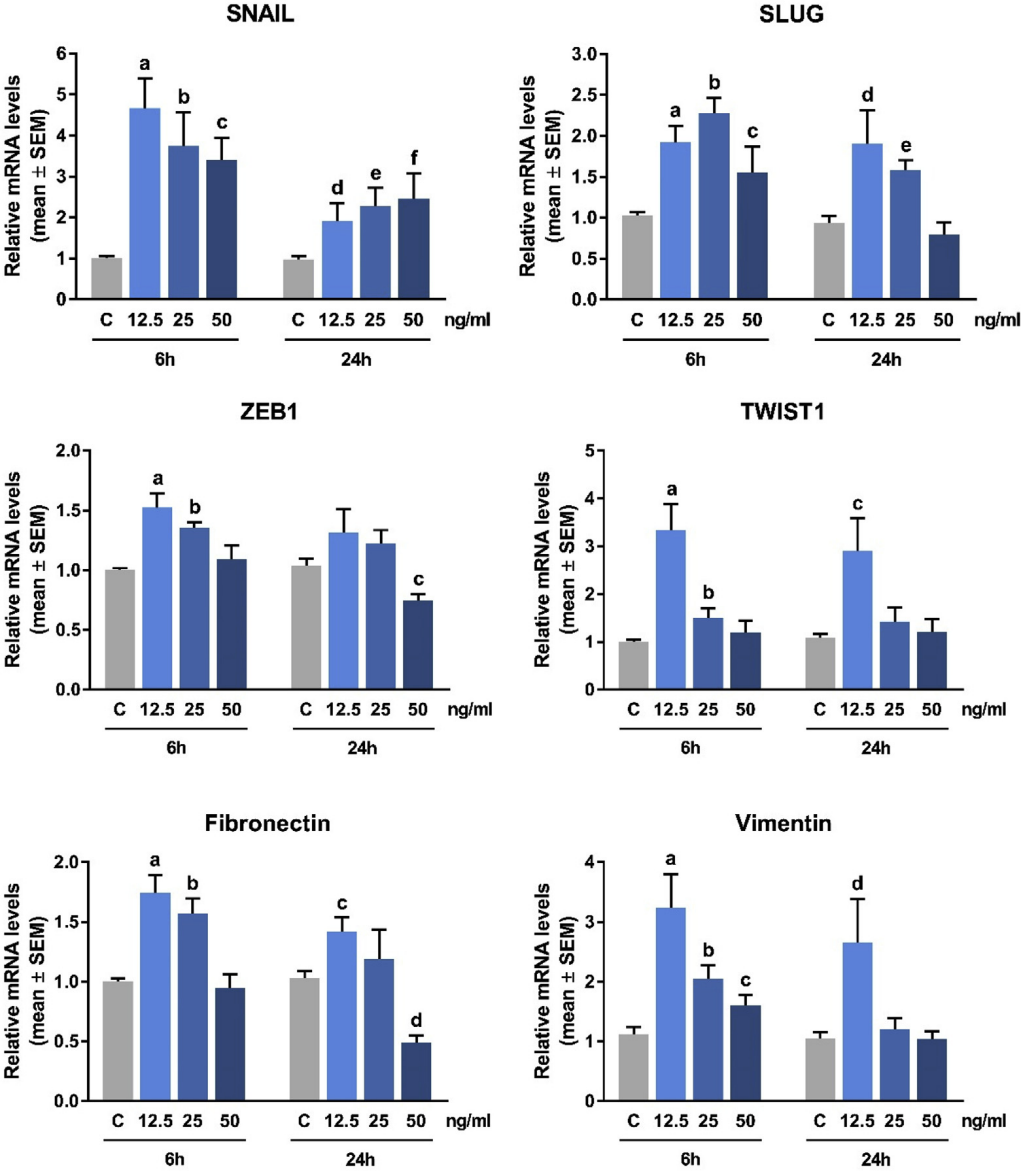
**Effect of resistin on mesenchymal gene expression in MDA-MB-231 cells.** Quantitative RT-PCR analyses data on the relative mRNA expression levels of SNAIL, SLUG, ZEB1, TWIST1, fibronectin and vimentin in MDA-MB-231 cells, untreated or treated with resistin (12.5, 25, and 50 ng/ml for 6 and 24 hours). All fold-change data and *p* values are listed in Table 2.

Using similar treatment conditions, we evaluated the effect of resistin on the expression of various mesenchymal and epithelial markers in MCF-7 and MDA-MB-231 cells. Results from qRT-PCR analysis showed that in MDA-MB-231 cells, resistin significantly upregulated mRNA levels of key transcription factors involved in EMT (SNAIL, SLUG, ZEB1, and TWIST1) as well as their target genes, fibronectin and vimentin (Fig. 2 and Table 2). Results from Western blot analyses using MCF-7 and MDA-MB-231 cells demonstrated that resistin significantly increased the protein expression levels of SNAIL and vimentin in both MCF-7 and MDA-MB-231 cells, as well as concomitantly suppressed the protein levels of E-cadherin and claudin-1 in MCF-7 cells ([3] and Table 3). In order to investigate EMT transcription factor nuclear translocation events, we further performed fractioned Western blot, analyzing ZEB1 and SNAIL protein expression in separate cytoplasmic and nuclear fractions from MCF-7 and MDA-MB-231 cells treated with resistin (12.5 ng/ml for 6 hours). The results from this experiment demonstrated that resistin time-dependently induced the nuclear translocation of these two transcription factors in both, MCF-7 and MDA-MB-231 cells ([3] and Table 4).

**Table 2:** **Effect of resistin on mesenchymal gene expression in MDA-MB-231 cells.** Concentration-response and time-dependent experiment for resistin treatment of MDA-MB-231 cells (see Fig. 2). Cells were treated with various concentrations of resistin (12.5, 25 and 50 ng/ml) for 6 and 24 hours and relative mRNA levels of SNAIL, SLUG, ZEB1, TWIST1, fibronectin and vimentin were measured by using qRT-PCR analysis. *p* values < 0.05 are shown in bold.

Resistin (ng/ml)		12.5		25		50		12.5		25		50	
Treatment Time (h)		6						24					
Gene	Condition	mean ± SEM	p Value	mean ± SEM	p Value	mean ± SEM	p Value	mean ± SEM	p Value	mean ± SEM	p Value	mean ± SEM	p Value
SNAIL	Control	1.015 ± 0.048						0.980 ± 0.079					
	Resistin	4.669 ± 0.721	**0.0006**	3.743 ± 0.823	**0.0027**	3.400 ± 0.549	**<0.0001**	1.922 ± 0.434	**0.0398**	2.271 ± 0.452	**0.0020**	2.457 ± 0.621	**0.0040**
SLUG	Control	1.025 ± 0.043						0.933 ± 0.094					
	Resistin	1.921 ± 0.205	**0.0019**	2.282 ± 0.185	**<0.0001**	1.553 ± 0.315	**0.0267**	1.909 ± 0.404	**0.0263**	1.580 ± 0.124	**0.0004**	0.797 ± 0.147	0.4247
ZEB1	Control	1.002 ± 0.016						1.036 ± 0.060					
	Resistin	1.526 ± 0.119	**0.0002**	1.357 ± 0.042	**<0.0001**	1.096 ± 0.111	0.3641	1.318 ± 0.193	0.1813	1.223 ± 0.113	0.1220	0.748 ± 0.054	**0.0014**
TWIST1	Control	1.009 ± 0.035						1.088 ± 0.075					
	Resistin	3.335 ± 0.549	**0.0008**	1.500 ± 0.209	**0.0108**	1.201 ± 0.243	0.4293	2.901 ± 0.684	**0.0245**	1.427 ± 0.301	0.2253	1.219 ± 0.261	0.6319
Fibronectin	Control	1.001 ± 0.025						1.034 ± 0.058					
	Resistin	1.742 ± 0.153	**0.0074**	1.573 ± 0.122	**0.0093**	0.947 ± 0.116	0.7563	1.420 ± 0.121	**0.0362**	1.191 ± 0.245	0.6319	0.492 ± 0.058	**0.0003**
Vimentin	Control	1.125 ± 0.121											
	Resistin	3.241 ± 0.560	**0.0006**	2.050 ± 0.226	**0.0014**	1.607 ± 0.175	**0.0289**	2.658 ± 0.732	**0.0337**	1.201 ± 0.189	0.5287	1.044 ± 0.122	0.9197

**Table 3:** **Effect of resistin on epithelial and mesenchymal proteins expression in MCF-7 and MDA-MB-231 cells – densitometry analysis.** MCF-7 and MDA-MB-231 cells were treated with resistin (12.5 ng/ml) for 6 and 24 hours. Densitometry analyses were performed using ImageJ software (NIH). Data are presented as % change of resistin treatment compared to control (no treatment) after normalizing to GAPDH levels. *p* values < 0.05 are shown in bold.

Treatment Time		6h		24h	
Protein	Condition	Protein Expression (% ± SEM)	p Value	Protein Expression (% ± SEM)	p Value
MCF-7					
SNAIL	Control	100.00 ± 0.660			
	Resistin	195.90 ± 20.090	**0.0002**	236.10 ± 27.660	**0.0002**
Vimentin	Control	100.00 ± 1.801			
	Resistin	147.10 ± 12.320	**0.0016**	262.70 ± 15.400	**< 0.0001**
E-cadherin	Control	100.00 ± 9.933			
	Resistin	82.59 ± 6.082	0.2093	55.42 ± 6.600	**0.0201**
Claudin-1	Control	100.00 ± 12.460			
	Resistin	84.19 ± 9.331	0.3672	58.65 ± 4.992	**0.0369**
MDA-MB-231					
SNAIL	Control	100.00 ± 21.420			
	Resistin	249.80 ± 26.860	**0.0005**	235.80 ± 21.660	**0.0004**
Vimentin	Control	100.00 ± 16.980			
	Resistin	201.00 ± 13.060	**0.0002**	170.70 ± 18.370	**0.0122**

**Table 4:** **Effect of resistin on EMT-transcription factor nuclear translocation in MCF-7 and MDA-MB-231 cells – densitometry analyses.** MCF-7 and MDA-MB-231 cells were subjected to resistin treatment (12.5 ng/ml) for 6 and 24 hours. Western blots were performed using separate (cytoplasmic and nuclear) protein fractions. β-Tubulin and lamin B1 were used as loading controls. Densitometry analyses were performed using ImageJ software (NIH). Data are presented as % change of resistin treatment compared to control (no treatment) after normalizing to the corresponding loading control. *p* values < 0.05 are shown in bold.

Protein	Fraction	Condition	MCF-7		MDA-MB-231	
			Protein Expression (% ± SEM)	p Value	Protein Expression (% ± SEM)	p Value
SNAIL	Cytoplasmic	Control	100.00 ± 13.380		96.67 ± 9.349	
		Resistin (12.5 ng/ml): 6h	55.09 ± 7.723	**0.0438**	58.63 ± 9.535	**0.0465**
		Resistin (12.5 ng/ml): 24h	40.23 ± 14.720	**0.0398**	61.88 ± 4.449	**0.0283**
	Nuclear	Control	100.00 ± 4.044		100.00 ± 8.182	
		Resistin (12.5 ng/ml): 6h	218.500 ± 20.000	**0.0044**	158.10 ± 11.900	**0.0159**
		Resistin (12.5 ng/ml): 24h	208.100 ± 9.646	**0.0004**	173.30 ± 13.670	**0.0100**
ZEB1	Cytoplasmic	Control	98.100 ± 7.932		93.33 ± 4.913	
		Resistin (12.5 ng/ml): 6h	54.61 ± 8.793	**0.0213**	46.16 ± 10.970	**0.0172**
		Resistin (12.5 ng/ml): 24h	46.39 ± 10.730	**0.0179**	37.42 ± 15.890	**0.0283**
	Nuclear	Control	100.00 ± 4.884		100.00 ± 13.350	
		Resistin (12.5 ng/ml): 6h	219.80 ± 6.828	**0.0001**	168.60 ± 15.550	**0.0287**
		Resistin (12.5 ng/ml): 24h	255.50 ± 7.281	**0.0179**	191.70 ± 8.551	**0.0044**

## 2 Experimental design, materials and methods

### 2.1 Reagents

Human recombinant resistin (Cat. # PF-138) was purchased from Calbiochem^®^ (EMD Millipore Corp.). The lyophilized form was reconstituted with water to a concentration of 100 μg/ml and further to a series of dilutions of 8.3, 12.5, 25, and 50 μg/ml, which were used for cell treatment.

### 2.2 Cell lines and reagents

Human breast cancer MCF-7 and MDA-MB-231 cells were purchased from American Type Culture Collection (ATCC) (Cat. ## HTB-22 and HTB-26, ATCC). Cells were grown in Dulbecco’s Modified Eagle’s Medium (DMEM) (Cat. # 10-013-CF, Corning Inc.) supplemented with 10% FBS (Cat. # 1500-500, VWR International) and 1x Antibiotic/Antimycotic Solution (Cat. # 30-004-CI, Corning, Inc.).

### 2.3 Quantitative RT-PCR analyses

Total RNA was extracted by using TRIzol™ reagent (Ambion, Thermo Fisher Scientific) and chloroform, and precipitated with iso-propanol (VWR International). The precipitated RNA was washed once with 70% ethanol, air-dried and resuspended in sterile, nuclease-free water, then the samples were incubated at 65 °C for 10 minutes and stored at −86 °C until analyzed. Total RNA concentration was determined using NanoDrop™ One Microvolume UV–Vis Spectrophotometer (Thermo Fisher Scientific), samples were normalized to 1 mg/ml RNA, and RT reaction was performed by using qScript cDNA Synthesis Kit (Quanta Bio) and SimpliAmp thermal cycler (Applied Biosystems, Thermo Fisher Scientific). Quantitative PCR analyses were performed by using PowerUp SYBR Green Master Mix and QuantStudio 3 Real-Time PCR System (Applied Biosystems, Thermo Fisher Scientific). The following primer sequences (5′-3′) were used: SOCS3, (forward) CCTGCGCCTCAAGACCTTC, (reverse) GTCACTGCGCTCCAGTAGAA (PrimerBank ID 45439351c1); SNAIL, (forward) AAGATGCACATCCGAAGCCA, (reverse) CAAAAACCCACGCAGACAGG [5]; SLUG, (forward) CTTCCTGGTCAAGAAGCA (reverse), GGGAAATAATCACTGTATGTGTG [6]; ZEB1, (forward) GCACAACCAAGTGCAGAAGA, (reverse) GCCTGGTTCAGGAGAAGATG [7]; TWIST1, (forward) GGCTCAGCTACGCCTTCTC, (reverse) CTAGTGGGACGCGGACAT [8]; fibronectin, (forward) ATGATGAGGTGCACGTGTGT, (reverse) CTCTGAATCCTGGCATTGGT [7]; vimentin, (forward) AGATGGCCCTTGACATTGAG, (reverse) TGGAAGAGGCAGAGAAATCC [7]; GAPDH, (forward) CCTGCACCACCAACTGCTTA, (reverse) GGCCATCCACAGTCTTCTGAG [9]. The annealing temperature for all of the PCR analyses was 60 °C.

### 2.4 Western blot analyses

Whole cellular protein was extracted by using Pierce™ RIPA Lysis Buffer (Cat. # 89901, Thermo Fisher Scientific) supplemented with protease inhibitor cocktail (Mammalian ProteaseArrest, Cat. # 786-331, G-Biosciences) and phosphatase inhibitors (PhosphataseArrest™, Cat. # 786-450, G-Biosciences). Cytoplasmic and nuclear protein fractures were extracted by using Nuclear & Cytoplasmic Extraction Kit (G Biosciences^®^, Cat. # 786-182) following manufacturer’s protocol. Protein concentrations were quantified by using Pierce™ BCA Protein Assay Kit (Cat. # 23225, Thermo Fisher Scientific), BioTek^®^ plate reader and Gen5™ data analysis software (BioTek^®^). Protein extracts were separated by polyacrylamide gel electrophoresis (SDS-PAGE), and transferred onto nitrocellulose membranes (Amersham, GE Healthcare). Membranes were blotted with SNAIL (C15D3) Rabbit mAb (Cat. # 3879), SLUG (C19G7) Rabbit mAb (Cat. # 9585), TCF8/ZEB1 (D80D3) Rabbit mAb (Cat. # 3396), Vimentin (D21H3) XP Rabbit mAb (Cat. # 5741), E-cadherin (24E10) Rabbit mAb (Cat. # 3195), Claudin-1 (D5H1D) XP Rabbit mAb (Cat. # 13255), GAPDH (14C10) Rabbit mAb (Cat. # 2118) (Cell Signaling Technology), β Tubulin (D-10) mAb (Cat. # sc-5274), and Lamin B1 (B-10) mAb (Cat. # sc-374015) (Santa Cruz Biotechnology) primary antibodies and Pierce™ Goat Anti-Rabbit Horseradish Peroxidase Conjugated Secondary Antibody (Cat. # 31460, Thermo Fisher Scientific). Protein bands were visualized by using SuperSignal West Pico Chemiluminescent Substrate (Cat. # 34580, Cell Signaling Technology) and MyECL™ Imager (Thermo Fisher Scientific). Densitometry analyses were performed with ImageJ software (National Institutes of Health) and protein expression levels were presented as percentage difference compared to control (±SEM).

### 2.5 Statistical analyses

Quantitative RT-PCR analyses data were based on 4-6 separate experiments, as each experimental condition was set up in duplicates, and each sample was analyzed in duplicates. Densitometry analysis of the Western blot data was based on 3 different experiments. Statistical analysis were carried out using GraphPad Prism 7 software (GraphPad Software, La Jolla, CA). Data were analyzed using ANOVA. Results are expressed as mean ± SEM and are considered statistically significant if *p* < 0.05.
